# K15 Protein of Kaposi’s Sarcoma Herpesviruses Increases Endothelial Cell Proliferation and Migration through Store-Operated Calcium Entry

**DOI:** 10.3390/v10060282

**Published:** 2018-05-24

**Authors:** Wei Chen, Changqing Xu, Liuqing Wang, Bing Shen, Linding Wang

**Affiliations:** 1 Department of Microbiology, Anhui Medical University, Hefei 230032, China; chenwei1413@163.com (W.C.); xcq521208@163.com (C.X.); 2 Department of Clinical Laboratory, the Third People’s Hospital of Hefei, Hefei 230051, China; Wangliuqinghf@126.com; 3 Department of Physiology, Anhui Medical University, Hefei 230032, China; shenbing@ahmu.edu.cn

**Keywords:** Kaposi’s sarcoma-associated herpesvirus, K15, store-operated calcium entry, Orail1, cell proliferation, cell migration

## Abstract

Kaposi’s sarcoma (KS) is a tumor of the vascular endothelium that is caused by Kaposi’s sarcoma-associated herpesvirus (KSHV). K15 of KSHV is a specific gene encoding a transmembrane protein. Two highly different forms of K15, the predominant (K15P) and minor (K15M) have been identified in different KSHV strains. In genomic locations and protein topology, two K15 alleles resemble the latent membrane protein (LMP) 1 and LMP2A of Epstein–Barr virus. Both K15 proteins have motifs similar to those found in LMP1 and LMP2A. K15 therefore seems to be a hybrid of a distant evolutionary relative of LMP1 and LMP2A. Ca^2+^ is a second messenger and participates in numerous activities in cells, like proliferation, migration and metastasis. It has been found previously that LMP1 increased Ca^2+^ influx through store-operated calcium channels and blockade of LMP1 reduced store-operated Ca^2+^ entry (SOCE). LMP2A has similar activity. So we sought to determine whether K15 had similar activity. We showed that K15P induced Ca^2+^ influx and enhanced expression of Orail1, which is a vital protein in SOCE, and overexpression of K15P improved cell motility. Mutant K15P did not show these activities in HEK-293T and EA.hy 926 cells. Our results showed that K15P increased cell proliferation and migration though SOCE and established a novel mechanism for the development of KS and KSHV-associated diseases.

## 1. Introduction

Cell motility plays an important role in many diverse biological processes ranging from embryogenesis to immune responses [1]. Abnormal activation of cell motility in natural or tumor cells is the primary cause of death in the majority of cancer patients [2]. The metastatic phenotype is a complicated process, and we usually define it as the metastatic cascade. This process includes several steps: the ability to break through local physical barriers such as the basement membrane; migration from the primary tumor to blood or lymphatic vessels; survival in the circulation; and invasion of distant tissues and establishment of metastatic lesions [1,3].

Kaposi’s sarcoma (KS) is a tumor with abnormal vascular proliferation, and is one of the most frequent acquired immune deficiency syndrome (AIDS)-related cancers and a major health threat in sub-Saharan Africa [4,5]. KS herpesvirus (KSHV), also known as human herpesvirus (HHV)-8, was first identified in KS tissues by Chang et al. with representational difference analyses [6,7,8]. Regarded as the etiological agent of KS, the virus was associated with two lymphoproliferative disorders: primary effusion lymphoma (PEL) and multicentric Castleman’s disease (MCD) [9,10]. In advanced KS lesions, one of the important proliferative elements is KSHV-infected endothelial cells, which lose their typical morphology, become spindle-shaped, and acquire invasive characteristics [11].

Open reading frame 75, K15, consists of eight exons that are alternatively spliced and encodes a putative transmembrane protein at the far right-hand end of the long unique coding region of the KSHV genome [12,13,14]. Two highly divergent forms of K15 have been identified: predominant (P) and minor (M) [12,14]. These two alleles have only 33% amino acid identity and yet K15 protein is predicted to feature 12 transmembrane segments and a putative cytoplasmic signal-transducing carboxyl terminus, which contains several putative signaling motifs such as two SH2-binding sites (Y^431^ASIL and Y^481^EEVL), a proline-rich SH3-binding site (P^387^PLP), and a tumor necrosis factor receptor associated factor (TRAF)-binding site (A^473^TQPTDD) [14,15,16,17]. Previous studies have demonstrated that K15 protein interacts with cellular proteins such as members of the Src family of protein tyrosine kinases and TRAFs via its C-terminal domain, thereby activating mitogen-activated protein kinases like MAPK4, c-jun N-terminal kinase (JNK) 1, and extracellular signal-regulated kinase 2, as well as the nuclear factor (NF)-κB pathway, resulting in activation of activator protein (AP)-1 and nuclear factor of activated T-cells (NFAT)-dependent gene expression [18]. K15-induced activation of gene expression in cells was dependent on Y481 of the SH2-binding site, when using the mutant of K15 (the Y481 to F481), the expression of gene was reduced [19]. Microarray studies have also revealed that K15 upregulates the expression of genes involved in angiogenesis and cell migration. So, when these genes are detected in epithelial cells, K15 induces the production of several proteins that are known to be involved in cell motility [20,21].

Epstein–Barr virus (EBV) or HHV-4 also belongs to the *Gammaherpesvirinae* and is an important etiological agent of nasopharyngeal carcinoma (NPC) [22]. Latent membrane proteins (LMPs) encoded by EBV have been identified as major pathogen factors in the development of EBV-related human cancers [23,24]. LMP1 and LMP2A enable EBV-infected cells with diverse malignant properties to participate in the process of malignancy [23,24,25]. In genomic locations and protein topology, two K15 alleles resemble the LMP1and LMP2A of EBV. K15 has a genomic location and predicted protein structure like that of LMP2A [26]. Both K15 proteins have motifs similar to those found in EBV LMP1 and LMP2A, because the C terminus of K15 has sequences similar to those found in EBV LMP1, including a putative TRAF-binding site [18,27]. K15 therefore seems to be a hybrid of a distant evolutionary relative of EBV LMP1 and LMP2A [26,28]. Thus, with so many similar characteristics with K15 and LMP1, LMP 2A, or KSHV and EBV, we were convinced that K15, LMP1, and LMP2A have analogical functions when the viruses infect cells and cause related diseases.

In many types of cells, intracellular store depletion of Ca^2+^ causes an influx of extracellular Ca^2+^ through store-operated calcium entry (SOCE) [29,30]. Previous studies have shown that LMP1 of EBV increases Ca^2+^ influx through SOCE [31]. In contrast, when LMP1-modulated SOCE is impeded, calcium influx is reduced in NPC and cell migration is inhibited [32]. SOCE is mediated via specific plasma membrane channels in response to the depletion of intracellular Ca^2+^ stores. This Ca^2+^ entry pathway is a common and omnipresent mechanism regulating Ca^2+^ influx into cells [33]. SOCE consists of two necessary proteins, stromal interaction molecule (STIM) 1 and Orail1, respectively. STIM1 is a single transmembrane protein on the endoplasmic reticulum (ER) membrane and Orail1 is a four-transmembrane domain protein on the plasma membrane. The N terminus of STIM1 is located in the lumen of the ER and senses the depletion of luminal Ca^2+^. The C terminus of STIM1 is located in the cytosol and activates SOCE upon store depletion by coupling to Orail1 [29,31,32,33]. K15 resembles LMP1 and LMP2A in protein structure and has the same ability to promote cell migration and proliferation, but the mechanism is not clear. Whether K15 also increases cell proliferation and migration via SOCE remains unknown.

In summary, human *Gammaherpesvirinae* have been shown to promote cell migration and invasion [18,21,24]. KSHV promotes invasion of primary human umbilical vein endothelial cells by inducing matrix metalloproteinases and AP-1 pathway [34]. EBV also has the function by upregulating the expression of genes and signaling pathways. In this study we found that the modulation of calcium influx by K15 contributed to cell proliferation and motility via SOCE. We also showed that overexpression of K15 enhanced formation of Orail1, which is a vital membrane protein of SOCE. Our findings may establish a novel mechanism and contribute to KSHV-induced cell migration and KS tumor metastasis studies.

## 2. Materials and Methods

### 2.1. Cell Culture

HEK-293T cells and human endothelium-derived cell line, EA.hy926, were purchased from American Type Culture Collection (Manassas, VA, USA) and cultured in Dulbecco’s modified Eagle’s medium (DMEM) supplemented with 10% fetal bovine serum (FBS), penicillin at 100 U/mL, and streptomycin at 100 μg/mL (Invitrogen, Carlsbad, CA, USA) at 37 °C in a 5% CO_2_-humidified atmosphere.

### 2.2. Plasmids and Transfections

The pFJ-EA, pFJ-K15P, and pFJ-K15P (Y^481^F), the mutants of K15P, were constructed previously in our laboratory [14,18]. The four plasmids, pCDH-CMV-GFP, pMDL-PRRE, pRSV-Rev, and pMD2.g, were a kind gift of Professor Shen (Anhui Medical University, Hefei, China). Two recombinant plasmids, K15P and K15P (Y^481^F), were generated by polymerase chain reaction (PCR) amplification of pFJ-K15P and pFJ-K15P (Y^481^F) with forward primer (5′-AGATTCTAGAGCTAGCGAATGAAGACACTCATATTCTTCT-3′) and reverse primer (5′-CCATAATTCGCTAGCTGTTCCTGGGAAATAAAACCTCC-3′). The resulting PCR product was cloned into pCDH-CMV-GFP and sequenced. The lentivirus of K15P and K15P (Y^481^F) was constructed using pCDH-CMV-K15P-GFP, pCDH-CMV-K15P (Y^481^F)-GFP, pMDL-PRRE, pRSV-Rev and pMD2.g. For transfection, HEK-293T cells were grown to subconfluency and transfected using Lipofectamine 2000 (Invitrogen).

### 2.3. Production and Purification of Lentiviral Virions

To produce lentiviral virions of K15P and K15P (Y^481^F), pCDH-CMV-K15P-GFP, pCDH-CMV-K15P (Y^481^F)-GFP, pMDL-PRRE, pRSV-Rev, and pMD2.g were co-transfected into HEK-293T cells (2 × 10^5^/mL; 60 mL total). After 6 h, the transfection medium was replaced with 10 mL fresh DMEM plus 10% FBS and the cells were cultured for 3 days before harvesting the virus. To purify the virus, cell culture supernatant (60 mL) was collected, followed by low-speed centrifugation (3000× *g*; 10 min) to remove cells and cellular debris. The supernatant was then subjected to high-speed centrifugation (28,000× *g*) at 4 °C for 2 h. The pellet was resuspended in 6 mL basic DMEM without supplements, and the purified and concentrated viral stock solution was used to infect EA.hy926 cells. One milliliter of purified viral solution of K15P and K15P (Y^481^F) was used to infect 5 × 10^5^ EA.hy926 cells for 3 days, followed by determining the percentage of green fluorescent protein (GFP)-positive cells under a fluorescence microscope. For mock infection, equivalent numbers of HEK-293T cells were subjected to the same viral induction and purification procedures, and the resulting pellet was resuspended in the same amount of basic DMEM and used for control infection.

### 2.4. Measurement of Cytosolic Ca^2+^

HEK-293T cells transfected with pFJ-EA, pFJ-K15P, and pFJ-K15P(Y^481^F) or EA.hy926 cells infected with lentiviral vector, lentivirus K15P(lenti-K15P), and lentivirus K15P (Y^481^F)[lenti-K15P (YF)] were seeded on circular glass coverslips in 1.5 mL DMEM for 24 h and the cells were 70–80% confluent before measurement. At the time of the experiment, the cells were loaded with 10 μmol/L Fluo-8/AM at 37 °C for 30 min. Ca^2+^ stores were depleted by treating cells with 4 μmol/L thapsigargin (TG) for 10 min in Ca^2+^-free phosphate-buffered saline (PBS), which contained 140 mmol/L NaCl, 5 mmol/L KCl, 1 mmol/L MgCl_2_, 10 mmol/L glucose, 0.2 mmol/L EGTA, and 5 mmol/L HEPES, pH 7.4. Ca^2+^ influx was initiated by applying 2 mmol/L extracellular Ca^2+^. Fluorescence was recorded using a Leica TCS SP5 confocal laser system (Heidelberg, Germany). Changes in cytosolic Ca^2+^ [Ca^2+^]_I_ were displayed as the ratio of fluorescence relative to the intensity before the application of extracellular Ca^2+^ (F1/F0).

### 2.5. Western Blotting

Proteins were extracted from the lysates of HEK-293T or EA.hy926 cells with lysis buffer that contained 1% Nonidet P-40, 2 mM EDTA, 150 mmol/L NaCl, and 50 mmol/L Tris-HCl, pH 7.4, with complete protease inhibitor. All western blotting was performed according to standard protocols, using SDS-PAGE, polyvinylidene difluoride (PVDF) membranes, and 5% nonfat milk in PBS–Tween for blocking. The PVDF membrane carrying transferred proteins was incubated at 4 °C overnight with the primary antibodies: anti-GFP, anti-STIM1, or anti-Orail1 (Santa Cruz Biotechnology, Santa Cruz, CA, USA; 1:200 dilution). Immuno detection was accomplished using horseradish peroxidase-conjugated secondary antibody, followed by processing through an enhanced chemiluminescent (ECL) detection system. The optical density of each blot was normalized to that of glyceraldehyde 3-phosphate dehydrogenase (GAPDH) analyzed within the same lane and represented as relative optical density.

### 2.6. Cell-Counting Kit-8 (CCK-8) Assay and Wound Scratch Assays

HEK-293T cells transfected with pFJ-EA, pFJ-K15P, and pFJ-K15P (Y^481^F) or EA.hy926 cells infected with lentiviral vector, lentivirus K15P (lenti-K15P), and lentivirus K15P (Y^481^F) [lenti-K15P (YF)] were seeded in 96-well plates at 10^4^–10^5^ cells in one well with 100 μL DMEM plus 10% FBS, and were cultured in a CO_2_ incubator at 37 °C for 24 h. Twenty-four hours later, 100 μL fresh complete medium replaced the old medium and 10 μL cell-counting kit-8 (CCK-8) reagents were added to each well. The plate was incubated at 37 °C for 2 h and absorbance at 450 nm was measured using a PECTRA max 190 reader (molecular device, San Jose, CA, USA). HEK-293T or EA.hy926 cells transfected and infected with three types of plasmids or lentiviruses, as described above, were grown in six-well plates to near confluence. The monolayers were wounded at 2 days post-infection with a plastic pipette tip, and cellular debris was removed by washing with aseptic PBS. Bright-field images (Nikon E400, Tokyo, Japan) were obtained at 0, 12, and 24 h. The cell migration rate was calculated by IPWIN60 software (Media Cybernetics, Inc., Rockville, MD, USA).

### 2.7. Statistical Analysis

Statistical analysis was performed using Sigmaplot software (12.0, IBM, Armonk, NY, USA). All graphical values were represented as the mean ± standard error. The *t*-test was used for statistical analysis. *p* < 0.05 was considered as statistically significant.

## 3. Results

### 3.1. K15P Amplified Thapsigargin-Stimulated SOCE in HEK-293T and EA.hy926 Cells

**Figure 1:**
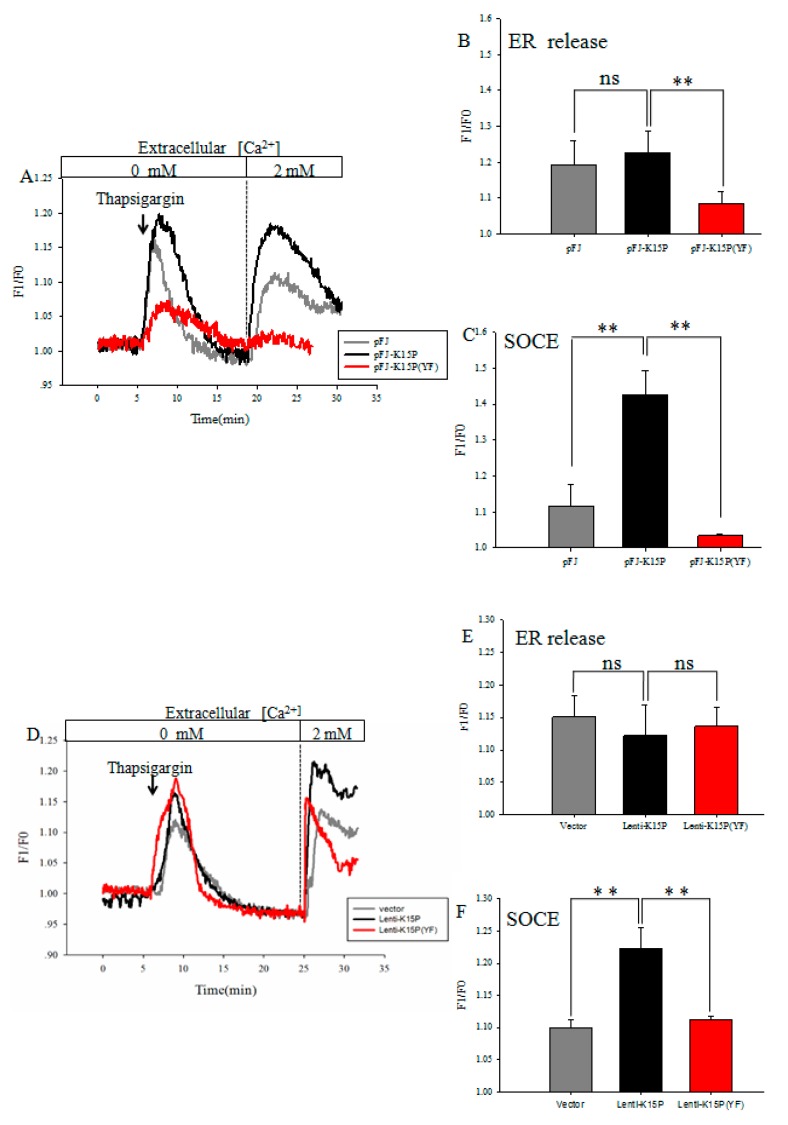
Store-operated calcium entry (SOCE) in HEK-293T and EA.hy926 cells. (**A**) Changes in cytosolic Ca^2+^ concentration ([Ca^2+^]_cyto_) were measured in HEK-293T cells. Representative traces showing Ca^2+^ release in Ca^2+^-free saline solution by Ca^2+^-indicator fura-8 and 2 mM Ca^2+^ application-induced [Ca^2+^]_I_ rise. (**B**) Histogram in the right panel shows the average peak from the baseline after thapsigargin-induced depletion of ER Ca^2+^ in HEK-293T cells. (**C**) Summary of data showing increase in [Ca^2+^]_I_ in response to extracellular Ca^2+^ application after thapsigargin in HEK-293T cells. (**D**) Changes in [Ca^2+^]_cyto_ were measured in EA.hy926 cells. (**E**) Histogram in the right panel shows the average peak from the baseline after thapsigargin-induced depletion of ER Ca^2+^ in EA.hy926 cells (**F**) Summary of data showing changes in [Ca^2+^]_I_ in response to extracellular Ca^2+^ application after thapsigargin in EA.hy 926 cells. *n* = 5 per group (the *n* refers to technical replicates). Data are presented as the mean ± standard error. ** *p* < 0.01.

We studied whether K15P amplified thapsigargin-stimulated SOCE in two cell lines. TG was used to activate SOCE by passive depletion of internal Ca^2+^ stores from the ER. The two cell lines were treated with 4 μM thapsigargin in Ca^2+^-free saline solution for ~20 min, after which 2 mM Ca^2+^ was added to the extracellular solution. TG-evoked Ca^2+^ release was significantly induced in the HEK-293T cells transfected with K15P compared with pFJ and K15P (YF) (Figure 1A,C). Compared with the cells infected with Lenti-K15P and vector or Lenti-K15P (YF), we obtained the same results in EA.hy926 cells (Figure 1D,F). Consistently, with the different treatments in two cell lines, K15P had no effect on ER-released Ca^2+^, but significantly boosted the subsequently evoked SOCE (Figure 1B,E).

### 3.2. Expression of STIM1 and Orail1in HEK-293T and EA.hy926 Cells

**Figure 2:**
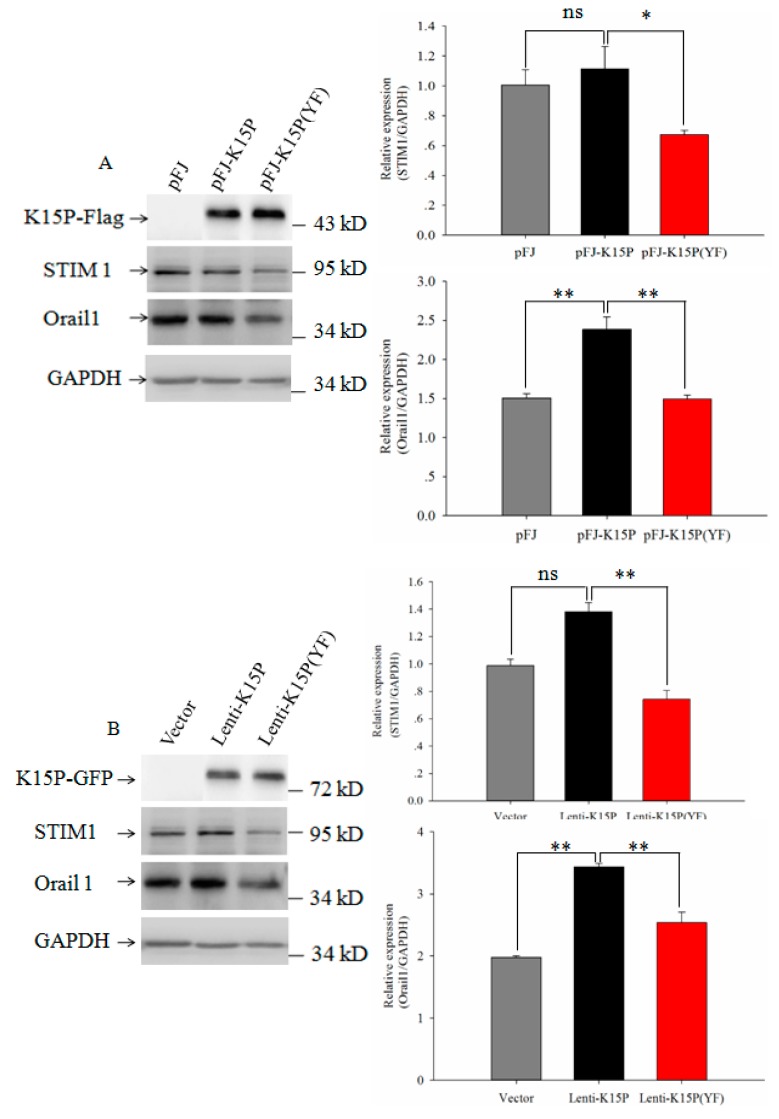
Expression of STIM1 and Orail1 in the two cell lines. (**A**) Western blots of K15P, STIM1, and Orail1 expression in HEK-293T cells. The right panel shows expression of STIM1 and Orail1 quantified by densitometric analyses. (**B**) Western blots of K15P, STIM1, and Orail1 expression in EA.hy926 cells. The right panel shows expression of STIM1 and Orail1.GAPDH was used as a control. Data are presented as the mean ± standard error. *n* = 5 per group. ns, not significant. ** *p* < 0.01, * *p* < 0.05.

STIM 1 is a single transmembrane protein on the ER membrane, is located in the lumen of the ER, and senses the depletion of luminal Ca^2+^. Orail1 is a four-transmembrane domain protein on the plasma membrane. The C terminus of STIM1 is located in the cytosol and activates SOCE upon store depletion by coupling to Orail1. Many studies have demonstrated that STIM1 and Orail1 proteins are involved in SOCE. To study the mechanism for the promotion by K15P via SOCE, we examined the expression levels of the STIM1 and Orail1 proteins in HEK-293T and EA.hy926 cells. Western blotting showed that expression of STIM1 was unchanged in HEK-293T and EA.hy926 cells in the group of pFJ and K15P. However, compared with the group of K15P and K15P (YF), there was significantly reduced expression of STIM 1 (Figure 2A). In the group of K15P, expression of Orail1 was significantly increased compared with other groups (Figure 2B).

### 3.3. K15P Increased Cell Proliferation and Migration

**Figure 3:**
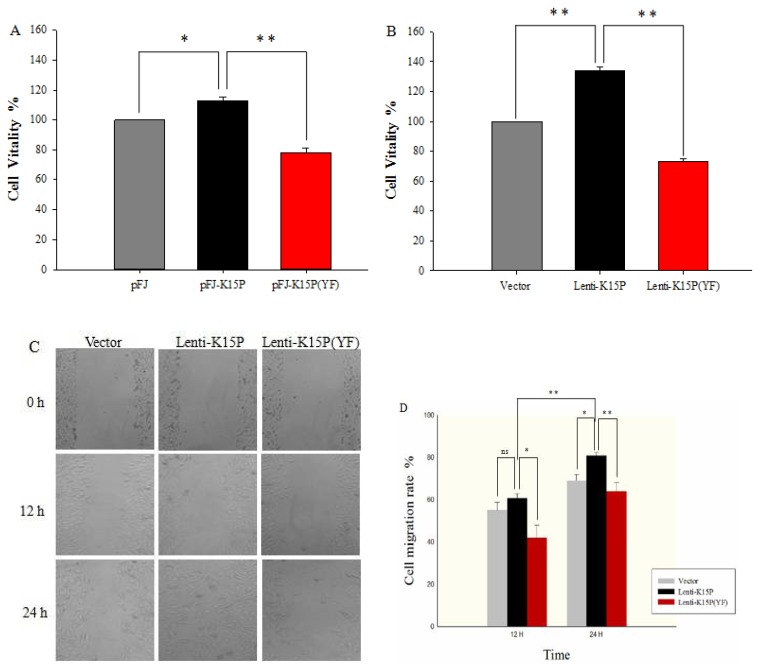
Cell proliferation and migration determined by CCK-8 assays and wound scratch assays. (**A**) Percentage cell viability with different treatments in HEK-293T cells. (**B**) Percentage cell viability with different treatments in EA.hy926 cells. (**C**) Representative photographs of migratory cells were captured at three times point after scratching (100×). (**D**) The right panel shows that cell migration was quantified by measuring the maximum migration distance with different treatments at indicated times in EA.hy926 cells. Data are presented as the mean ± standard error. *n* = 3 per group (the *n* refers to technical replicates). ns, not significant. ** *p* < 0.01, * *p* < 0.05.

Many studies have demonstrated that SOCE plays a vital role in cell proliferation and migration. K15P also promotes cell proliferation and migration. We investigated whether K15P promoted cell proliferation and migration via SOCE. CCK-8 assays were used to assess cell proliferation and the wound scratch assay was used to evaluate cell migration. K15P notably increased cell proliferation compared with the other two groups. Mutant K15P, K15P (YF), significantly reduced cell proliferation (Figure 3A,B). Wound scratch assays showed that K15P promoted cell migration compared with the other groups. On the contrary, K15P (YF) inhibited this ability of K15P (Figure 3C,D).

## 4. Discussion

KSHV is the pathogen of KS, which is a vascular tumor with pronounced inflammatory histological features that arises from infected endothelial cells [9,35]. Among all the stages of KS, aberrant angiogenesis and cell migration are the most important characteristics [11]. Previous studies have shown that KSHV can induce the formation of capillary junctions in infected cells on Matrigel in cultured primary endothelial cells [28]. In advanced lesions, KSHV-infected endothelial spindle cells predominated and were thought to represent the neoplastic component of this tumor [7,8,35]. KS lesions may regress following treatment of human immunodeficiency virus with antiretroviral therapy, or moderating iatrogenic immune suppression, especially in transplant recipients, indicating that this tumor requires the continued presence of KSHV and the expression of viral genes [36,37]. KSHV and EBV both belong to the *Gammaherpesvirinae*. KS resembles other virus-driven proliferative diseases such as EBV-associated post-transplant lymphoproliferative disease [26].

It is known that intracellular Ca^2+^ is a critical second messenger linking external stimuli to cell functions [29]. If the cell is not capable of maintaining an adequate level of Ca^2+^ in intracellular stores, many cellular functions including post-translational modifications of recombinant proteins will be abnormal [31]. Ca^2+^ signaling plays a critical role in almost every aspect of cellular functions and enables cells to adapt to external environmental changes [38]. In the progression of infection by KSHV, Ca^2+^ exerts an important influence at all stages [39]. Previous studies have shown that changes in the intracellular Ca^2+^ concentration mediate angiontensin-2 release and Ca^2+^ chelators and Ca^2+^ channel blockers inhibit angiontensin-2 release. Angiotensin-2 has been identified as a vital element in the growth of early-stage KS and the cytokine proangiogenic or proinflammatory processes [40]. The growth of all-stage KS depends on various growth factors, chemokines, and cytokines. K15 gene, a part of the KSHV genome, also has the same function to promote cells to release chemokines and activate the downstream signaling pathways [12,39,40,41]. The YEEV motif in K15P is the target for members of the family of Src tyrosine kinases, and phosphorylation of this motif is required for activation of the MEK/ERK2, JNK, and NF-κB pathways by K15P in epithelial cells [14,15,16,17,19]. K15-induced activation of gene expression in cells was dependent on Y^481^ of the SH2-binding site, when using the mutant of K15 (the Y^481^ to F^481^), the expression of the gene was reduced [13,14,15,16,17,19]. In EBV and KSHV, LMP1 or LMP2A and K15 have many similar structures and functions. Therefore, K15 seems to be a hybrid of a distant evolutionary relative of LMP1 and LMP2A [26,28]. On the basis of previous data, it has been found that LMP1 increased calcium influx through store-operated channels in B lymphoid cells, and blockade of LMP1-modulated SOCE reduced metastatic potential in NPC [31,32]. Therefore, the relationship between LMP1 and SOCE may influence cell activation, proliferation, and migration. However there are no reports that K15 can promote cell proliferation and migration via SOCE.

**Figure 4:**
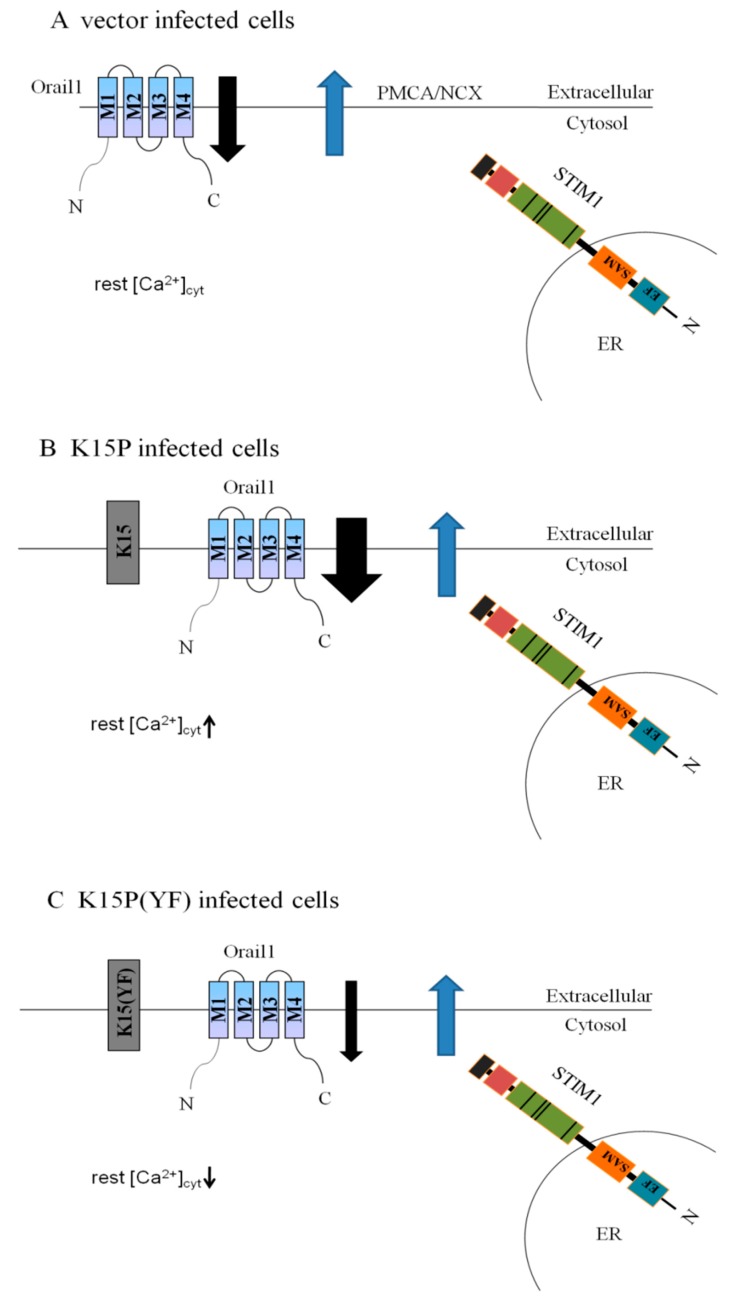
Proposed mechanism of K15-induced disturbance of Ca^2+^ influx. (**A**) In the control group with vector infection, [Ca^2+^]_cyt_ was in equilibrium between Ca^2+^ entry by SOCE and Ca^2+^ exit by plasma membrane Ca^2+^ ATPases (PMCA) and the Na^+^/Ca^2+^ exchanger (NCX). (**B**) In K15P-infected cells, K15P was expressed, inducing an increase of SOCE and an increase of Ca^2+^ influx. The residual [Ca^2+^]_cyt_ levels increased compared with the control and K15P (YF) groups. (**C**) In K15P (YF)-infected cells, the mutant protein induced an decrease of SOCE and Ca^2+^ influx, and the residual [Ca^2+^]_cyt_ levels did not increase compared with other group.

In our study, the use of different K15P genes provided a complex picture and showed a possible pathway to explain that K15P can increase calcium ion influx through SOCE. When Ca^2+^ stores are depleted with thapsigargin, STIM1 combines with Orail1 to open store-operated calcium channels and allow Ca^2+^ influx (Figure 4A). At the same time, on the plasma membrane, plasma membrane Ca^2+^ ATPases (PMCA) and/or Na^+^/Ca^2+^ exchanger (NCX) probably act to effuse intracellular Ca^2+^. The expression of K15P increases Ca^2+^ influx, so we hypothesize that when K15P expression increases, the combination of STIM1 and Orail1 also increases and more channels are opened on the plasma membrane, thus the residual [Ca^2+^]_cyt_ levels increase (Figure 4B). Western blotting showed that the expression of Orail1, which is an important component of SOCE, increased compared with other proteins. After K15P (YF) was transfected into the cells, the expression of Orail1 was reduced, which would lead to the decreasing of SOCE, so we hypothesize that the combination of STIM1 and Orail1 is reduced, and we can also conclude that the motif of YEEV is essential for increase of Ca^2+^ influx by the K15P protein (Figure 4C).

Previous studies have demonstrated that SOCE participates in Ca^2+^ signaling and is upregulated in cell proliferation and migration [31,32,38]. In our study, K15P promoted cell proliferation and migration through SOCE and upregulated the expression of Orail1. We also found that the motif of YEEV was vital for the K15P/STIM1/Orail1 pathway. However, another partner, K15M, has been identified as a divergent form of K15. It is not possible to conclude whether K15M has the same function in the process of KSHV infection. Besides STIM1 and Orail1, other isoforms including STIM2, ORAI2, and ORAI3 are also widely expressed in mammalian cells. Compared with STIM1, STIM2 can weakly activate ORAI and is more sensitive to small changes in ER Ca^2+^ concentration [42]. Recent studies have shown that Orail1, ORAI2, and ORAI3 are all expressed in the kidneys [43]. However, the expression patterns and functional roles of STIM2, ORAI2, and ORAI3 in endothelial cells were unknown in the present study. Therefore, we cannot exclude these components to function in SOCE and in the proliferation or migration of endothelial cells. Future studies of the roles of STIM2, ORAI2, and ORAI3 should clarify the whole picture.

In summary, our study shows for the first time a link between K15P and SOCE in KSHV infection in endothelial cells and we hypothesize that the K15P/STIM1/Orail1 pathway plays a critical role in KS metastasis, on the basis of its ability to drive cell proliferation and migration. Our findings may reveal a novel mechanism of K15P induction of cell proliferation and migration, which might elucidate a new pathway in the tumor development of KS.
